# A travelable area boundary dataset for visual navigation of field robots

**DOI:** 10.1038/s41597-025-04457-3

**Published:** 2025-01-28

**Authors:** Kai Zhang, Xia Yuan, Jiachen Xu, Kaiyang Wang, Shiwei Wu, Chunxia Zhao

**Affiliations:** 1 School of Computer Science and Engineering, Nanjing University of Science and Technology, Nanjing, 210000 China; 2 Software Development Department, Dahua Technology, Hangzhou, 310000 China

**Keywords:** Computer science, Scientific data

## Abstract

Travelable area boundaries not only constrain the movement of field robots but also indicate alternative guiding routes for dynamic objects. Publicly available road boundary datasets have outlined boundaries by binary segmentation labels. However, hard post-processes have to be done to extract from detected boundaries further semantics including the shapes of the boundaries and guiding routes, which poses challenges to a real-time visual navigation system without detailed prior maps. In addition, boundary detectors suffer from insufficient data collected from complex roads with severe occlusion and of different shapes. In this paper, a travelable area boundary dataset is semi-automatically built. 82.05% of the data is collected from bends, crossroads, T-shape roads and other irregular roads. Novel guiding semantics labels, shape labels and scene complexity labels are assigned to boundaries. With the support of the new dataset, travelable area boundary detectors could be trained, evaluated and fairly compared. The dataset can also be used to train, evaluate or test detectors for the road boundary detection task.

## Background & Summary

Travelable area boundaries as inherent elements of roads, can be reliably used for navigation^1^ and self-localization^2,3^ without additional assistance such as road markings or even detailed prior maps, which is essential in places where prior guidance is absent, for instance, parks and campuses. Road boundary detectors are applied to structured roads with stable differences in elevation, material or gradient between travelable areas and other functional areas^4^. Three-dimensional (3D) point clouds are used since accurate distance measurement under different weather conditions^5,6^ allows geometric properties such as gradients^7,8^ and height information^9^ to be easily obtained. Complex scenes still pose challenges to perception. As one of the common issues, the impact of occlusion has been paid attention and alleviated^6,10^, especially with the advancements of deep neural networks^11^, though. Due to the lack of targeted dataset collected from roads with severe occlusion and of different shapes, performance is affected, especially in bends and intersections^12^.

**Fig. 1:**
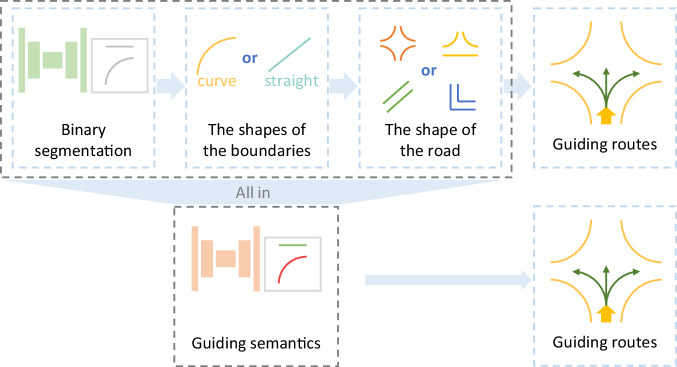
A post process of perception to planning after detection. The first row shows some steps carried out after a road boundary detector detecting binary boundaries. The shapes of the detected boundaries and the shape of the road should be recognized. Finally, alternative guiding routes are obtained. The second row illustrates that guiding semantics provides the shape information of concern in a more direct way, which lightens the burden of the post process. The yellow arrows represent the position and direction of the robot.

Binary segmentation labels provided by road boundary datasets^9,11,13^ outline and locate boundaries. They are the results of perception but far away from planning. After getting boundaries, as illustrated in Fig. 1, the shapes of the boundaries can be obtained by calculating and analyzing the gradients between adjacent curbs. Then the shape of the road can be obtained by analyzing the relationships among the boundaries. Recognizing the shapes of boundaries and roads is beneficial for field robots to move smoothly. Finally, guiding routes are available according to path planning. Such a post process should be elaborate so that further semantics, for instance, the shapes of boundaries or the shapes of roads could be timely fed into the next stage in a real-time visual navigation system without detailed prior maps. Fortunately, with the help of powerful feature extractors based on deep neural networks, some steps, such as recognizing the shapes mentioned above could be skipped and omitted from the process of perception to planning, as long as there is a dataset providing corresponding semantic labels.

**Fig. 2:**
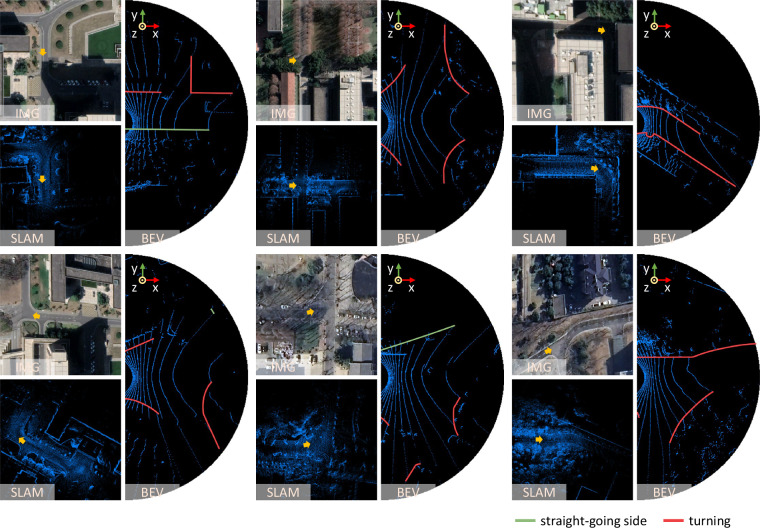
Typical scenes in the TAB^14^. Satellite images from the Google Earth (https://earth.google.com) and point cloud maps^17^ of the entire scenes are attached. The yellow arrows represent the position and direction of the robot. The bird’s eye view of the corresponding point cloud is displayed to the right of each group. Crossroads, T-shape intersections, right-angled bends and other irregular roads are included in the TAB^14^. Cars, pedestrians, cyclists, barriers and traffic cones are common objects and recorded.

To fill the gaps mentioned above, a travelable area boundary dataset (TAB^14^) is built in a semi-automatic way. Point clouds are collected in our campus and 82.05% of them are from bends and intersections. Scenes with multiple objects were carefully and specially selected. As illustrated in Fig. 2, not only straight roads, crossroads, T-shape roads, but also some uncommon roads of irregular shapes are recorded. Cars, pedestrians and cyclists, on the other hand, as common dynamic objects in the campus and cities^15,16^, are recorded in our dataset. They move either from far to near or vice versa. They gather in groups or columns. Some static objects such as parked cars, bicycles, barriers and traffic cones^16^ are also recorded. Objects may be crowded or sparse. On average, there are 13 objects in each consecutive point cloud sequence. And the maximum number of objects in a sequence can reach 51.

Contrary to the methods devoting to improving perception and planning algorithms^18^, the TAB^14^ tackles the issue from a data-centric perspective. Boundaries are defined and reflect the semantics of roads in the TAB^14^. They are outlined in the way similar to the binary segmentation labels provided by road boundary datasets^9,11,13^. In addition, guiding semantics labels are assigned to the boundaries to narrow the gap between perception and planning since they infer the shapes of boundaries and roads and provide driving suggestions, as illustrated in Fig. 1. A boundary is labeled as turning, which means there is a bend or an intersection and robots and other dynamic objects are allowed or able to change their routes and turn with a high probability. A boundary is labeled as a straight-going side recommending robots to go straight along the boundary. Compared to the shapes of boundaries, guiding semantics can stably predict and claim in advance the shapes of boundaries and the shapes and trends of roads. Therefore, the provided annotations not only outline roads but also refer the shapes and trends of roads, and furtherly give alternative routes to robots and other dynamic objects.

Diverse and poorly maintained roads, interactions among robots and objects, and the sparsity of point clouds jointly enhance the complexity of scenes, which usually causes a boundary that needs to be observed to be partially or completely lost in the collected point clouds and hence not only poses challenges to the perception of robots, but also increases the difficulty and uncertainty of annotation. Scene complexity labels are proposed to indicate these affected parts of boundaries and assess the impact of the complexity of scenes. They directly play an important role during evaluation. Moreover, if these scene complexity labels can be put to good use in the training phase, powerful and efficient supervision could be accomplished.

The TAB^14^ provides a total of 42 consecutive point cloud sequences, in which 6,350 frame point clouds along with 15,315 boundaries are labeled. Table 1 shows publicly available road boundary datasets along with our TAB^14^. Due to the emphasis on bends and intersections where interactions and collisions are prone to occur, compared to the NRS^13^ containing similar number of point cloud frames, the TAB^14^ collects far more frames from bends and intersections (5,210 frames vs about 2k frames).

**Table 1:** Publicly available road boundary datasets and the TAB^14^.

Dataset (year)	Frames	Boundaries	Binary segmentation	Shape	Guiding semantics	Scene complexity
VeCAN^9^ (2018)	200	—	*√*	—	—	—
Uncertainty^11^ (2021)	5,224	—	*√*	—	—	—
NRS^13^ (2023)	6,220	12.4k	*√*	—	—	—
TAB^14^ (2024)	6,350	15.3k	*√*	*√*	*√*	*√*

In recent years, the binary segmentation labels of boundaries can be extracted from semantic segmentation datasets^19,20^ by automatic or semi-automatic methods^21,22^ since boundaries are lines between roads and other functional areas. However, guiding semantics cannot be well extracted from semantic segmentation labels. Due to dynamic changes and interactions, the time-sensitive guiding semantics of the same boundary in two adjacent frames may be different. Therefore, a semi-automatic annotation method is carried out. Firstly, ground points are extracted by point cloud ground segmentation methods^23,24^. Then coarse curbs can be obtained as they are the points between the ground and non-ground areas. Since some objects in sparse point clouds may be difficult to recognize for annotators, the curbs serve as cues and indicate the potential boundaries.

In summary, our primary contribution is manifold.

- A novel travelable area boundary dataset is built in a semi-automatic way. It collects 3D point clouds from roads with severe occlusion and of different shapes. As an extended road boundary dataset, it supports training and evaluating detection models for complex scenes.
- The proposed guiding semantics explores the role of boundaries in visual navigation without detailed prior maps for field robots. It reduces the post process after detection and the gap from perception to planning in a data-centric perspective and enables boundaries to directly give alternative routes to robots and other dynamic objects so that visual navigation systems could be more efficient.
- The parts of boundaries affected by the diversity of boundaries, the interactions among objects and the sparsity of point clouds are assessed and taken into account during evaluation to alleviate the impact on perception and annotation. Moreover, the proposed scene complexity labels indicating the affected parts can facilitate precise detection if they are put in good use during training.
- A new visual task, travelable area boundary detection, is proposed that not only boundaries should be detected but also their guiding semantics should be predicted. The provided well-labeled data with evaluation metrics supports the research on the proposed task.

## Methods

**Fig. 3:**
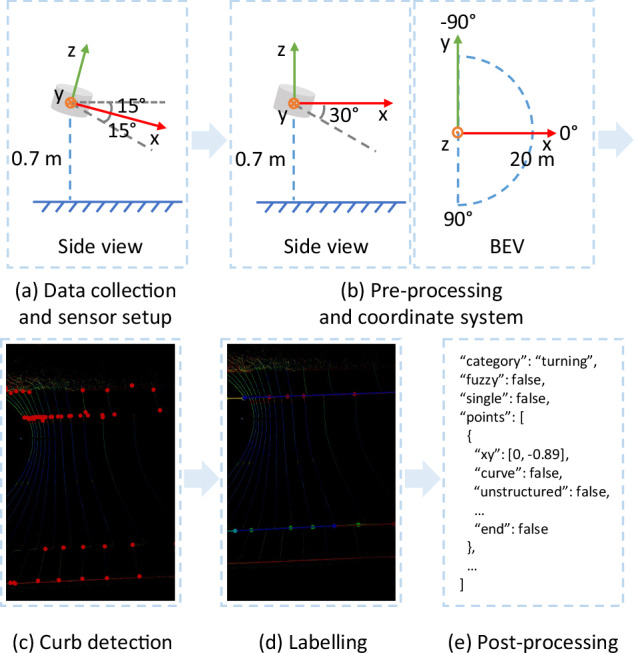
The process of building the TAB^14^.

As illustrated in Fig. 3, the process of building the dataset includes collecting data, pre-processing point clouds, automatically labelling curbs, manually labelling and post-processing. In the pre-processing stage, the coordinate system is adjusted and the bird’s eye views (BEVs) of point clouds are prepared for labelling. Coarse curbs detected by a curb detection method indicate potential boundaries, which is auxiliary for annotators. The open-source graphical image annotation tool, LabelMe (https://www.labelme.io), is used when labelling. There are various labels, including binary segmentation labels, guiding semantics labels, shape labels and scene complexity labels. In the post-processing stage, ground truth files are created. All data is split into a training set, a validation set and a test set so that detectors can be trained, evaluated and fairly compared. As an important part of the TAB^14^, evaluation metrics are illustrated in details.

### Sensor setup

A LiDAR (Light Detection and Ranging), Velodyne VLP-16, is used for data collection. The Velodyne VLP-16 is a 16-beam spinning long-range LiDAR with a 360-degree horizontal field of view (FOV) and a 30-degree vertical FOV. Its horizontal resolution is set to 0.2 degrees and vertical resolution is 2 degrees.

As illustrated in Fig. 3(a), the LiDAR is tilted forward on the top of a field robot with limited load capacity. The tilt angle in the vertical direction *θ* is -15 degrees, which optimizes the LiDAR’s front FOV to minimize the blind area in front of the LiDAR and focus on the area below the top of the robot, particularly the ground. The LiDAR is about 0.7 meters above the ground.

### Pre-processing

Given a raw point cloud *P*_*raw*_ = {(*x*_*m*_, *y*_*m*_, *z*_*m*_, *intensity*_*m*_, *beam*_*m*_)|0≤*m* < *N*_*raw*_}, where (*x*_*m*_, *y*_*m*_, *z*_*m*_) is the 3D coordinates of the point *p*_*m*_ ∈ *P*_*raw*_, *intensity*_*m*_ is the reflection intensity of the point *p*_*m*_ ∈ *P*_*raw*_, *beam*_*m*_ is the index of beam that the point *p*_*m*_ ∈ *P*_*raw*_ belongs to and *N*_*raw*_ is the number of the points that make up the point cloud *P*_*raw*_, the coordinate system of the point cloud *P*_*raw*_ is adjusted through a rotation transform *R* firstly so that, in theory, the XOY plane of the adjusted point cloud *P*_*adj*_ is parallel to the ground, as illustrated in Fig. 3(b). Given the 3D coordinates (*x*_*raw*,*m*_, *y*_*raw*,*m*_, *z*_*raw*,*m*_) of the point *p*_*m*_ ∈ *P*_*raw*_, the adjust coordinates are

1 $$\begin{array}{l}{({x}_{adj,m},{y}_{adj,m},{z}_{adj,m})}^{T}=R\times {({x}_{raw,m},{y}_{raw,m},{z}_{raw,m})}^{T}\\ R=\left[\begin{array}{ccc}1 & 0 & 0\\ 0 & \cos (-\theta ) & -\sin (-\theta )\\ 0 & \sin (-\theta ) & \cos (-\theta )\end{array}\right].\end{array}$$

Considering the sparsity and visibility of point clouds and the tradeoff between computational load and task requirements, the region of interest is within *ρ* = 20 meters in front of the robot and between −1.5 meters and 1.5 meters in the vertical direction, which preserves 89.48% of the points ahead in average. The final point cloud *P* ⊆ *P*_*adj*_ provided by the TAB^14^ is $P=\{({x}_{n},{y}_{n},{z}_{n},intensit{y}_{n},bea{m}_{n})| \sqrt{{x}_{n}^{2}+{y}_{n}^{2}}\, < \,\rho ,$ $-\pi /2\le \arctan ({y}_{n}/{x}_{n})\, < \,\pi /2,-1.5\le {z}_{n}\, < \,1.5,0\le n\, < \,N\le {N}_{raw}\}$, where *N* is the number of the points that make up the point cloud *P*.

BEVs of point clouds are generated by Algorithm 1 so that the point clouds can be annotated as images. The sizes of the BEVs are 2200 × 4200 where the paddings are 100 pixels on the four sides so that points located at the edge of the region of interest can be well observed and labeled. Therefore, points in a 0.01 × 0.01 × 3  *m*^3^ pillar are sampled and the highest point is picked out. The pixel value of the BEVs is the pseudo color calculated according to the reflection intensity of the highest point in the corresponding pillar. Obviously, compared to the resolutions of the LiDAR, the pillar is small enough so that the labelling is at centimeter level.

#### Algorithm 1.

Generating a bird’s eye view (BEV).

### Curb detection

Annotation is conducted with the assistance of curbs which are detected and automatically labeled by a curb detection method. Ground points are extracted firstly by the fast point cloud ground detection method^23^. Then curbs are detected because they are between ground and non-ground points. Detectors^25,26^ based on deep neural networks are usually trained using dense point clouds from LiDARs with 64 or more beams^16,19,20^. They are not used here due to the point clouds collected from the 16-beam LiDAR are too sparse for them to perform well. After adjusting parameters, methods^23,24^ based on well-designed manual features could apply to the sparse point clouds and quickly meet the requirements of semi-automatic annotation.

Curbs are projected onto the BEVs and presented as points. Annotation files are automatically initialized and record these curb points. Therefore, these curb points can be shown as elements in the LabelMe.

### Labelling

#### Binary segmentation labels

Polygonal lines, line segments and points are recommended to be used to manually outline boundaries. Therefore, binary segmentation labels indicating the positions of boundaries can be obtained according to these line-shape geometric figures. It is the primary goal for travelable area boundary detectors to detect boundaries which restrict the travelable areas of field robots.

#### Guiding semantics labels

Although some high-level semantics can be explored by analyzing the shapes of boundary lines, travelable area boundary detectors are expected to directly output these advanced semantics in an end-to-end way.

**Fig. 4:**
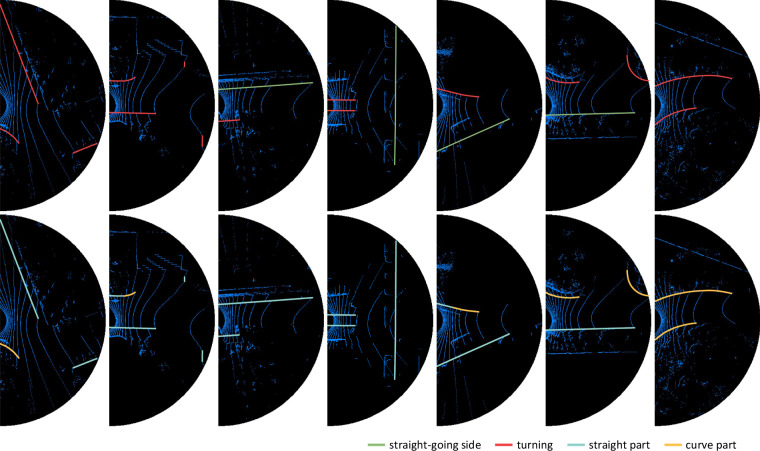
Guiding semantics and shapes. The first line is the semantics, and the second line is the corresponding shapes. The shapes of some boundaries may be straight, but there are turnings which indicate bends or intersections. Guiding semantics can stably predict and claim in advance the shapes of boundaries and the trends of roads.

As illustrated in Fig. 4, guiding semantics labels are assigned to outlined boundaries. There are two types of guiding semantics labels:

- A turning infers a bend or an intersection here or ahead where robots and other dynamic objects are allowed or able to change their routes and turn with a high probability. A boundary is assigned as a turning if 1) the shape of the boundary line is curve, 2) the visible ground surface is fan-shaped; 3) or the opposite boundary on the same side is present and visible.
- A straight-going side recommends robots had better go straight along the boundary. A boundary is assigned as a straight-going side if it does not meet any of the requirements for being a turning.

Detectors should predict the guiding semantics of boundaries along with the positions. A few boundaries are difficult to be accurately judge their semantics especially when a straight-going side turns into a turning. They are marked as fuzzy (0.15% of labeled boundaries) along with guiding semantics labels. During evaluation, these fuzzy boundaries are expected to be detected and located while their guiding semantics, regardless of which it is predicted to be, have no effect on the evaluation results.

#### Shape labels

The shape of a boundary line is one of criteria for the guiding semantics of the boundary as mentioned before. As illustrated in Fig. 4, a boundary could consist of straight parts or curve parts. Given a logical function *IF*() which checks whether the part is a specified attribute, curve parts are labeled as *IF*(*curve*) = *true*. Although detectors are not expected to analyze the shape of a boundary any more, shapes play a role in evaluation metrics.

#### Scene complexity labels

**Fig. 5:**
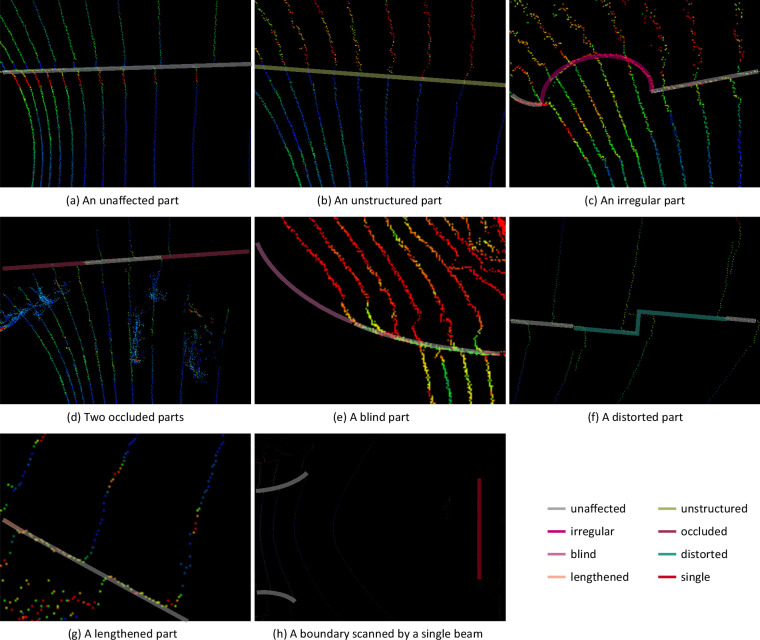
Examples of scene complexity labels.

**Fig. 6:**
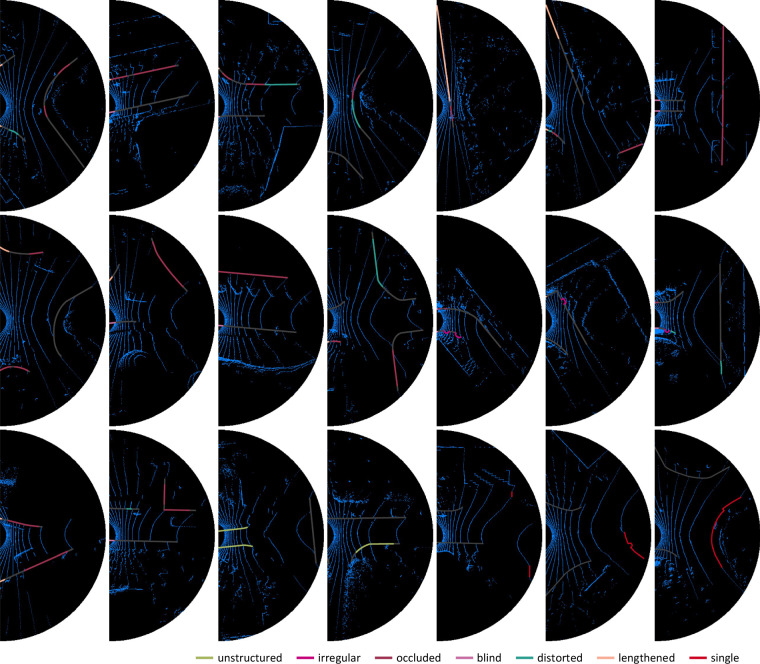
Scene complexity labels and affected parts.

Cars, pedestrians and other objects are everywhere in the real world, especially in parks and campuses. Complex scenes pose challenges to detectors and annotators. The TAB^14^ assesses the influence of the complexity of scenes on observation and annotation, focusing on the diversity of boundaries, the interactions between the robot and objects, and the sparsity of point clouds. A boundary is divided into several parts here. Each part may be labeled with one or more scene complexity labels. Figure 5 shows various types of scene complexity labels. Polygonal lines, line segments and points are recommended again to be used to outline the affected and labeled parts as illustrated in the Fig. 6.

**Diverse boundaries**. For a boundary itself, its structure and regularity are assessed:

- A structured road usually consists of curbs, fences and buildings which at least have a stable difference from the travelable area^4^. Boundaries of structured roads are almost structured. However, some parts of them may be unstructured due to damage, absence and vegetation coverage. On the contrary, boundaries of unstructured areas are almost unstructured. Unstructured parts are difficult to be located and outlined due to the loss of stable structure as illustrated in Fig. 5(b). They are labeled as *IF*(*us*) = *true*.
- As illustrated in Fig. 5(c), some parts are irregular due to manhole covers^4^ leading to boundary lines being not smooth. Path planning algorithms should avoid guiding robots into these irregular and protruding areas which are actually travelable but not appropriate. Therefore, it is acceptable that detectors ignore these protruding areas and imprecisely locate these irregular parts. These irregular parts are labeled as *IF*(*ir*) = *true*, which loosens the criteria for determining the correctness of prediction during evaluation.

**Interactions**. The interactions between the robot and dynamic and static objects are inevitable.

- Occlusion is a common impact on the observation of boundaries, which has been emphasized by prior works^6,10–12^. It is caused by the objects locating between the robot and boundaries. As a result, the occluded parts are lost in the collected point clouds and the affected boundaries are not continuous. Annotators have to fill in the occluded parts based on adjacent frames, which brings about annotation error. Figure 5(d) illustrates the effect of occlusion caused by some parked bicycles on the observation of a boundary. Occluded parts are marked and labeled as *IF*(*occ*) = *true* so that the annotation error can be taken into account during evaluation.
- Blindness of a LiDAR also results in the lack of observation and annotation error, which is caused by the LiDAR’s FOV and the relative position between the robot and boundaries. As illustrated in Fig. 5(e), blind parts are always closest to the robot, which affects the relative position between the robot and boundaries and threatens the safety of the robot. Blind parts are marked and labeled as *IF*(*bl*) = *true*.
- Point cloud distortion^27,28^ is an important issue in autonomous driving. Due to the working principle of spinning LiDARs, the points in a point cloud frame are not collected at the same time actually and the movement of LiDARs exacerbates the distortion. The faster a LiDAR moves, the more severe the distortion will be. As shown in Fig. 5(f), the boundary should have been straight but the ghost caused by distortion makes it discontinuous. A point in the real-world space is recorded twice in a point cloud frame and has two different coordinates. The two coordinates are correct at different times,but they are confusing for annotators and detectors. Severe distorted parts cannot clearly indicate where the boundary is and are marked and labeled as *IF*(*dt*) = *true* in the TAB^14^. The annotators are required to reserve the minimum travelable area to guarantee safe driving. Distortion removing methods usually requires the use of other sensors such as inertial measurement units^27^, which comes at a cost. Travelable area boundary detectors are expected to remove the effect of distortion via training. On the other hand, the evaluation metrics discussed later show tolerance for imprecise prediction caused by distortion.

**Sparse point clouds**. Although point clouds are able to describe 3D space, their drawback poses challenges to observation. The farther away from the LiDAR, the sparser the point clouds. Sparsity makes it impossible to observe continuous boundaries, which leads to annotators and detectors having to complete the missing parts.

- Like blind parts, the closest parts of the boundaries may be missed and should be lengthened and completed. These lengthened parts are labeled as *IF*(*len*) = *true*.
- Distant boundaries may only be scanned by a single beam which causes the locations of them are not accurate enough. These boundaries are labeled as *IF*(*single*) = *true*.

As mentioned above, the effects on observation are various. They may affect the same part. And it is hard to measure the weight of them. To simplify labelling, irregular parts, occluded parts, blind parts, distorted parts, lengthened parts are defined as being mutually exclusive. They are referred to as unclear parts, that is

2 $$IF(uc)=IF(ir)\,\,or\,\,IF(occ)\,\,or\,\,IF(bl)\,\,or\,\,IF(dt)\,\,or\,\,IF(len).$$

### Post-processing

After labelling guiding semantics, shapes and complexity, all labels are projected to the 3D point cloud space and share the coordinate system with the point cloud *P*. The *i*-th boundary *B*_*i*_ has been labeled with guiding semantics, a boolean value indicating whether its guiding semantics is fuzzy and a boolean value *IF*(*single*)_*i*_ indicating whether it is only scanned by a single beam.

For the *i*-th boundary *B*_*i*_, dense two-dimensional (2D) points are sampled every 0.01 meters along the X and Y axes. For the *j*-th point *d*_*i*,*j*_, shape and scene complexity labels are assigned to it if it belongs to certain affected parts. In addition, if a dense point is closest to the end of a boundary line, it is identified as an endpoint and labeled as *IF*(*end*)_*i*,*j*_ = *true*. Generally, a boundary line has two endpoints.

### Split

In the TAB^14^, continuous point cloud frames are organized in the form of sequences. In order to promote the research on the travelable area boundary detection, all labeled point cloud sequences are split into a training-validation set and a test set in an approximate 7:3 ratio. About 25% of the ground truth files in the training-validation set are randomly sampled as the validation set. Table 2 shows some statistics regarding the training-validation set and the test set.

**Table 2:** Statistics regarding the training-validation set and the test set.

	Training-validation set	Test set
Sequences	29	13
Frames	4418	1932
Bends and intersections	3688	1522
Boundaries	10664	4651

### Evaluation metrics

A boundary is consist of a set of points and a scene includes some boundaries. The expected prediction result is a set of points with guiding semantics. A set of predicted points with a certain guiding semantics will be tried to match with ground truth boundaries with the same guiding semantics and fuzzy boundaries no matter which guiding semantics labels they have.

Since there are correlations among boundaries, often manifesting as parallel or intersecting and appearing in pairs, travelable area boundary detectors are assessed at point level and boundary level during evaluation. The point-level assessment evaluates the completeness of each boundary while the boundary-level assessment evaluates the completeness of a scene.

**Keypoints**. To reduce computational consumption, dense points are sampled by BEV sampling as illustrated in Algorithm 2. The size of the BEV is 64 × 128. Therefore, for each boundary, keypoints with an interval of about 0.3 meters are prepared for evaluation, which are not sparse for autonomous driving tasks. The interval is larger than the tolerant radii of 99.6% of keypoints that will be illustrated below, which makes the offset prediction significant. In detail, for each 0.3125 × 0.3125  *m*^2^ grid, a keypoint is generated by aggregating dense points projected in the grid. The coordinates of it are the average of the coordinates of all sampled points. Other attributes of the keypoint are identified as long as these attributes exist in the sampled dense points.

#### Algorithm 2.

Generating keypoints.

**Tolerant radius**. Each keypoint has a tolerant radius which is affected by some factors including scene complexity labels. The predicted points within the radius are correctly predicted points. Meanwhile, the corresponding keypoint is recalled. A tolerant radius *r*_*i*,*k*_ of the *k*-th keypoint *g*_*i*,*k*_ belonging to the *i*-th boundary *B*_*i*_ is comprised of four parts.

- The base value *r*_*base*_ is 0.1 meters.
- If the keypoint is an endpoint, the radius increases by 0.1 meters.
- Since the working principle of the spinning LiDAR, the sparsity of point clouds is positively correlated with the distance from the origin. Therefore, the distance from the keypoint to the origin is a weight used to measure the sparsity.
- Taking scene complexity into consideration, the number of affected parts the keypoint *g*_*i*,*k*_ belongs to is a weight used to measure the complex impact on the observation of the keypoint. It is denoted as *μ*_*i*,*k*_.

For the *i*-th boundary *B*_*i*_, the set of keypoints is *G*_*i*_ = {(*x*_*i*,*k*_, *y*_*i*,*k*_, *IF*(*curve*)_*i*,*k*_, *F*(*us*)_*i*,*k*_, *IF*(*ir*)_*i*,*k*_, *IF*(*occ*)_*i*,*k*_, *IF*(*bl*)_*i*,*k*_, *IF*(*dt*)_*i*,*k*_, *IF*(*len*)_*i*,*k*_, *IF*(*end*)_*i*,*k*_)|0 ≤ *k* < *N*_*key*,*i*_}, where *N*_*key*,*i*_ is the number of the keypoints. The tolerant radius *r*_*i*,*k*_ of the *k*-th keypoint *g*_*i*,*k*_ is

3 $$\begin{array}{l}{r}_{i,k}={r}_{base}+IF{(end)}_{i,k}\times {r}_{base}+{\lambda }_{i,k}\times {r}_{base}\\ {\lambda }_{i,k}=\left(0.5\times \frac{\sqrt{{x}_{i,k}^{2}+{y}_{i,k}^{2}}}{20}+1\right)\times {\rm{lg}}\,\left(9\times {\mu }_{i,k}+1\right),0\le {\lambda }_{i,k} < 2.36\\ {\mu }_{i,k}=IF{(curve)}_{i,k}+IF{(us)}_{i,k}+IF{(uc)}_{i,k}+IF{(single)}_{i},0\le {\mu }_{i,k}\le 4\\ IF{(uc)}_{i,k}=IF{(ir)}_{i,k}\,\,or\,\,IF{(occ)}_{i,k}\,\,or\,\,IF{(bl)}_{i,k}\,\,or\,\,IF{(dt)}_{i,k}\,\,or\,\,IF{(len)}_{i,k}.\\ \end{array}$$

In theory, the minimum of the tolerant radius is 0.1 meters and the maximum is less than 0.436 meters. However, statistic shows the maximum is less than 0.42 meters and the average radius is about 0.15 meters.

**Point-level assessment**. F1-score is used to evaluate the completeness of a predicted point set. For the boundary *B*_*i*_ and the keypoint set *G*_*i*_, given the number of predicted points *Q*_*point*,*i*_, the number of correctly predicted points *N*_*correct*,*i*_≤*Q*_*point*,*i*_, the number of keypoints *N*_*key*,*i*_ and the number of recalled keypoints *N*_*rec*,*i*_, the point-level F1-score *F*_*p*,*i*_ can be calculated:

4 $$\begin{array}{l}precisio{n}_{i}=\frac{{N}_{correct,i}}{{Q}_{point,i}}\\ recal{l}_{i}=\frac{{N}_{rec,i}}{{N}_{key,i}}\\ {F}_{p,i}=\frac{2\times precisio{n}_{i}\times recal{l}_{i}}{precisio{n}_{i}+recal{l}_{i}}.\end{array}$$

In practice, more than one boundary will be predicted at once. The point-level F1-scores between predicted boundaries and labeled boundaries are calculated. They are treated as similarity scores and fed into the Kuhn-Munkres algorithm^29,30^, an one-to-one matching method. Therefore, the final F1-scores of the boundaries are determined.

**Boundary-level assessment**. Three thresholds are set, which are 0.3, 0.5 and 0.8. For each threshold *τ*, the predicted boundaries who are matched with labeled boundaries and whose point-level F1-scores are larger than or equal to the threshold are true positive predicted boundaries. Therefore, given the number of predicted boundaries *Q*_*bound*_, the number of true positive predicted boundaries *N*_*TP*_ ≤ *Q*_*bound*_ and the number of labeled boundaries *N*_*bound*_, the boundary-level F1-score *F*_*τ*_ can be calculated:

5 $$\begin{array}{l}precisio{n}_{\tau }=\frac{{N}_{TP,\tau }}{{Q}_{bound}}\\ recal{l}_{\tau }=\frac{{N}_{TP,\tau }}{{N}_{bound}}\\ {F}_{\tau }=\frac{2\times precisio{n}_{\tau }\times recal{l}_{\tau }}{precisio{n}_{\tau }+recal{l}_{\tau }}.\end{array}$$

## Data Records

**Fig. 7:**
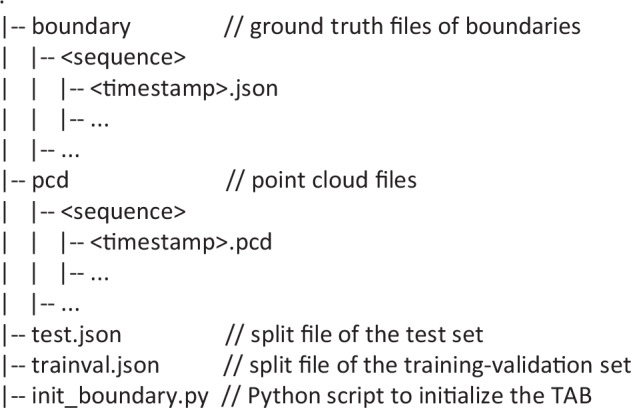
File structures of the TAB.

The dataset, TAB^14^, is available at the GitHub repository (https://github.com/kaiopen/tab). It offers point cloud files in PCD (Point Cloud Data) format, ground truth files in JSON (JavaScript Object Notation) format, split files in JSON format and a Python script to initialize the dataset. The point cloud files and ground truth files are named with timestamps and saved in different paths based on sequence numbers. They can be read as same as text files. Split files record sequence numbers and timestamps of point cloud files belonging to the training-validation set and the test set. All files are packaged and compressed, and available in the GitHub repository. Extract the files and ensure that the file structures are as shown in the Fig. 7. It is recommended to run the Python script before using the TAB^14^.

A Python toolkit for quick access to the TAB^14^ is provided in the GitHub repository (https://github.com/kaiopen/tab_kit). It provides the friendly interfaces to visit and visualize point clouds and labels. The evaluation methods are available, too.

## Technical Validation

### Statistics and characteristics

**Fig. 8:**
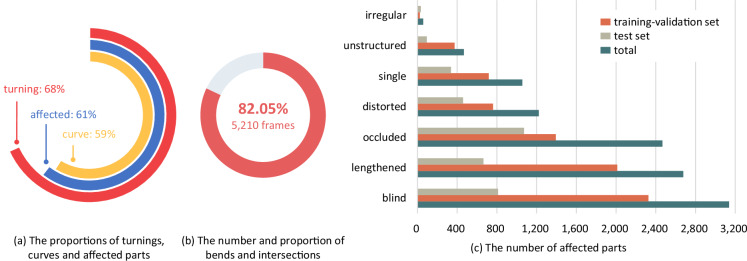
Statistics on guiding semantics, shapes and scene complexity.

The TAB^14^ provides 6,350 frame point clouds along with 15,315 labeled boundaries. As illustrated in Fig. 8(a), 59% of boundaries consist of curve parts and are curve while 68% are marked as turning, which proves that a turning may not a curve boundary. 0.15% of labeled boundaries are marked as fuzzy. And 52.17% of these fuzzy boundaries are straight-going sides. The proportion of fuzzy boundaries is very small. It is acceptable to ignore these fuzzy boundaries or ignore these fuzzy labels during training because the evaluation metrics are loose towards them.

**Emphasizing bends and intersections**. Interactions, including friction, usually occur at bends and intersections because turning is permitted. Hence, the TAB^14^ pays much attention to bends and intersections. As illustrated in Fig. 8(b), 82.05% scenes are bends and intersections.

**Assessing complex scenes**. The complexity of a scene with its impact on the observation of boundaries has been discussed before and labeled. As illustrated in the Fig. 8, 61% boundaries are affected.

**Fig. 9:**
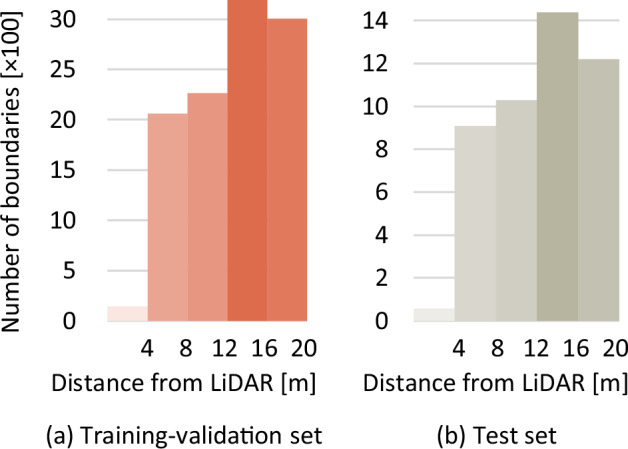
The farthest distances from the labeled boundaries to the LiDAR.

**Fig. 10:**
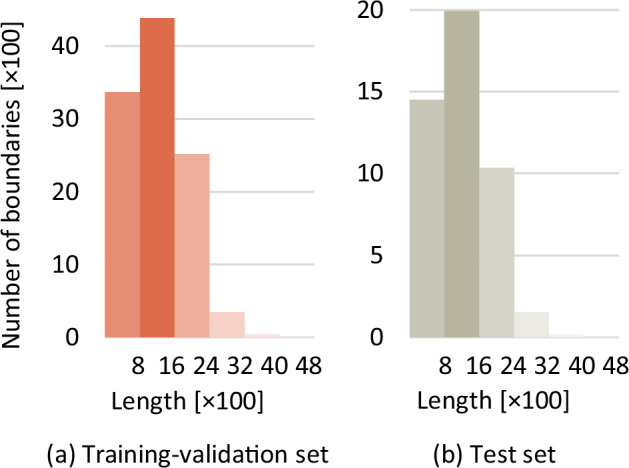
The lengths of the labeled boundaries, which are the numbers of dense points.

**Fig. 11:**
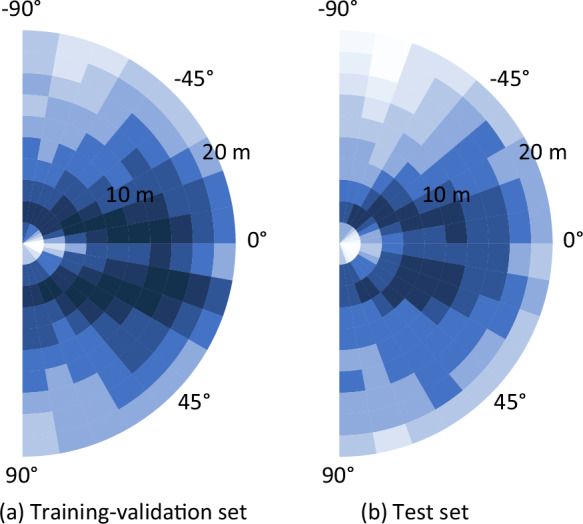
Spatial distributions of the labeled boundaries. Boundaries are usually located on both sides of the robot. There are also cases where some boundaries are in front of the robot, such as when the robot is turning around or driving at T-shape intersections.

**Comparable sets**. Although the training-validation set and the test set contain diverse scenes, the comparable statistics and distributions have been retained as illustrated in Figs. 9, 10 and 11.

### Efficiency of the semi-automatic annotation method

The significant amount of manpower expended on manual annotation is disapproved of. Therefore, a semi-automatic annotation method is used to improve the efficiency of annotation. Coarse curbs are detected by a curb detection method inspired by prior works^23,24^ according to the differences in elevation and gradient between travelable areas and other functional areas. These coarse curbs are expected to serve as cues and indicate the potential boundaries so that annotators could quickly identify boundaries and reduce omissions.

100 frame point clouds are randomly sampled from the TAB^14^ for the two efficiency experiments here. Eight participants are involved in the experiments. Four annotators have participated in the annotation and the other four participants are inexperienced and do not engaged in point cloud processing.

**Recognition accuracy**. It is primary for annotators to correctly identify and recognize boundaries so that accurate and precise annotation could be done, which can reduce the workload of verification and calibration. The eight participants are asked to identify boundaries, including pointing out their approximate locations and recognizing their shapes in each frame within 10 seconds. Table 3 reports the missing rate and recognition accuracy of the eight participants. During the experiment, distant and short boundaries were easily overlooked by participants, while coarse curbs could provided good cues. With the assistance of coarse curbs, the average missing rate of the four annotators has been decreased by 43.61% (4.93% vs 2.78%). Meanwhile, the average missing rate of the four inexperienced participants has been decreased by 53.30% (42.42% vs 19.81%) which is significant. In terms of recognizing boundaries, the average accuracy of the four annotators and the four inexperienced participants has been increased by 4.48% (89.91% vs 93.94%) and 27.59% (43.93% vs 56.06%) respectively. It can be seen that the coarse curbs can effectively reduce the missing rate and improve the recognition accuracy. For inexperienced participants, in particular, the missing rate has dropped dramatically. The assistance can effectively indicate to annotators where boundaries exist.

**Table 3:** Improvement in recognition accuracy.

	Missing rate (%) ↓		Recognition accuracy (%) ↑	
	Annotators	The inexperienced	Annotators	The inexperienced
Without coarse curbs	4.93	42.42	89.91	43.93
With coarse curbs	2.78	19.81	93.94	56.06

**Time efficiency**. Although annotations will be further modified during verification and calibration, annotators are expected to outline boundaries as precisely as possible. The four annotators are asked to outline boundaries as precisely as possible. Table 4 shows the time efficiency including the average time they consumed to complete annotating one frame and the average F1 scores of their annotations. Since coarse curbs not only avoid omissions but also eliminate some ambiguous annotations which have taken much time from the annotators, the average annotation time per frame has been reduced by up to 25.83% (49.02 seconds vs 36.36 seconds). Meanwhile, the annotations have become more precise.

**Table 4:** Improvement in time efficiency.

	SPF (s)	Straight-going side				Turning				Ignoring semantics			
		*mF*_*p*_	*F*_0.3_	*F*_0.5_	*F*_0.8_	*mF*_*p*_	*F*_0.3_	*F*_0.5_	*F*_0.8_	*mF*_*p*_	*F*_0.3_	*F*_0.5_	*F*_0.8_
Without coarse curbs	49.02	0.80	0.97	0.97	0.84	0.90	1.00	0.94	0.84	0.88	0.99	0.95	0.84
With coarse curbs	36.36	0.88	1.00	1.00	0.94	0.94	1.00	1.00	0.90	0.92	1.00	1.00	0.91

The SPF means seconds per frame. *mF*_*p*_ is the average point-level F1-scores.

### Performance among detectors

The UNet^31^, HRNet-w18^32^, DeepLabV3+^33^ are used as backbones to detect travelable area boundaries and verify the feasibility of the travelable area boundary detection task. The UNet^31^ is a typical semantic segmentation backbone used, especially in medical image processing. The HRNet-w18^32^ is a backbone for semantic segmentation and keypoint detection. The DeepLabV3+^33^ is an efficient convolution neural network for object detection and semantic segmentation. These backbones are combined with a pillar-based point cloud BEV encoder^34^ and two parallel heads. A multi-channel BEV image with a size of 256 × 512 is generated by the encoder and fed into the backbone. A heatmap and offsets^35^ constitute the prediction results after the tensor output by the backbone is respectively fed into the two heads. The sizes of the heatmap output by the UNet^31^ is the same as the input BEV while the sizes of the heatmaps output by the HRNet-w18^32^ and the DeepLabV3+^33^ are one quarter of the input BEV. All experiments use the same loss function, optimization algorithm and learning rate scheduler.

**Detecting keypoints**. Since boundaries are comprised of some keypoints during evaluation, the travelable area boundary detection is treated as a keypoint detection task^36^. The output heatmap has *C* channels, where *C* is the number of guiding semantics, and is of size *H* × *W*. Anchors are arranged densely and without overlap on the XOY plane of the point cloud space. One anchor corresponds to a pixel of the heatmap. Therefore, the heatmap models the probability of anchors being keypoints with specified semantics. In other words, given a keypoint *g*_*i*,*k*_ from the set of keypoints *G*_*i*_ of the *i*-th boundary *B*_*i*_ whose guiding semantics is *c*, its coordinates in the point cloud space is (*x*_*i*,*k*_, *y*_*i*,*k*_) and its coordinates in the heatmap space is (*u*_*i*,*k*_, *v*_*i*,*k*_) and

6 $$\begin{array}{l}{u}_{i,k}={x}_{i,k}/h\\ {v}_{i,k}=({y}_{i,k}-\rho )/w\\ h=\rho /H\\ w=(\rho -(-\rho ))/W,\end{array}$$

where *ρ* is the radius of the region of interest. For this keypoint *g*_*i*,*k*_, there is one ground-truth positive anchor located at (*u*_*i*,*k*_, *v*_*i*,*k*_) whose penalty ${\widehat{s}}_{(c,{u}_{i,k},{v}_{i,k})}=1$, and all other anchors are negative. During training, the penalty given to negative anchors within a radius of the positive anchor is reduced while the penalty of other anchors is 0. Here, the radius is equal to the tolerance radius *r*_*i*,*k*_ of the keypoint *g*_*i*,*k*_. Given an anchor located at (*u*, *v*) in the heatmap space, its distance ${d}_{(u,v)\to {g}_{i,k}}$ to the keypoint *g*_*i*,*k*_ located at (*x*_*i*,*k*_, *y*_*i*,*k*_) in the point cloud space is

7 $$\begin{array}{l}{d}_{(u,v)\to {g}_{i,k}}=\sqrt{{((u+0.5)\ast h-{x}_{i,k})}^{2}+{(((v+0.5)\ast w-\rho )-{y}_{i,k})}^{2}}\\ h=\rho /H\\ w=(\rho -(-\rho ))/W,\end{array}$$

and its penalty ${\widehat{s}}_{(c,u,v)}$ is

8 $${\widehat{s}}_{(c,u,v)}=\left\{\begin{array}{l}1,\,{\rm{if}}\,\,u=\lfloor {u}_{i,k}\rfloor \,\,{\rm{and}}\,\,v=\lfloor {v}_{i,k}\rfloor \\ {e}^{-\frac{{d}_{(u,v)\to {g}_{i,k}}^{2}}{2{\sigma }^{2}}},\,{\rm{if}}\,\,{d}_{(u,v)\to {g}_{i,k}} < {r}_{i,k}\\ 0,{\rm{otherwise}}.\end{array}\right.$$

Let *s*_(*c*, *u*, *v*)_ be the probability score at location (*u*, *v*) for guiding semantics *c* in the predicted heatmap, the variant of focal loss^35^ is

9 $${L}_{det}=-\frac{1}{K}\mathop{\sum }\limits_{c=0}^{C-1}\mathop{\sum }\limits_{u=0}^{H-1}\mathop{\sum }\limits_{v=0}^{W-1}\left\{\begin{array}{ll}{(1-{s}_{(c,u,v)})}^{\alpha }\log ({s}_{(c,u,v)}), & \,{\rm{if}}\,\,{\widehat{s}}_{(c,u,v)}=1\\ {(1-{\widehat{s}}_{(c,u,v)})}^{\beta }{s}_{(c,u,v)}^{\alpha }\log (1-{\widehat{s}}_{(c,u,v)}), & \,{\rm{otherwise}},\end{array}\right.$$

where *α* = 2 and *β* = 4 are the hyper-parameters which control the contribution of each anchor.

Predicting which anchor contains keypoints is only a rough indication of positioning. Hence the offsets *o*_(*c*,*u*,*v*)_ = (Δ*x*_(*c*,*u*,*v*)_, Δ*y*_(*c*,*u*,*v*)_) between the anchor and the ground truth keypoint should be predicted. Let ${\widehat{o}}_{(c,u,v)}=(\Delta {\widehat{x}}_{(c,u,v)},\Delta {\widehat{y}}_{(c,u,v)})=({u}_{i,k}-\lfloor {u}_{i,k}\rfloor ,{v}_{i,k}-\lfloor {v}_{i,k}\rfloor )$ be the ground truth offsets, the smooth L1 loss at positive anchors where ${\widehat{s}}_{(c,u,v)}=1$ is applied as illustrated in equation (10).

10 $$\begin{array}{l}SmoothL1(x,y)=\left\{\begin{array}{ll}0.5\times {(x-y)}^{2}, & \,{\rm{if}}\,\,| x-y|  < 1\\ | x-y| -0.5, & \,{\rm{otherwise}}\end{array}\right.\\ {L}_{off}=\frac{1}{K}\mathop{\sum }\limits_{k=0}^{K-1}<?RetainOpenmmlmfenced separators="" open="(" close=")"?>(SmoothL1(\Delta {x}_{(c,u,v)},\Delta {\widehat{x}}_{(c,u,v)})+SmoothL1(\Delta {y}_{(c,u,v)},\Delta {\widehat{y}}_{(c,u,v)})).\end{array}$$

**Results and analysis**. Table 5 shows the performance among the three models. Although the output of the UNet^31^ is more intensive than the other two, the F1 scores are not high. Most of boundaries have been predicted while the point-level F1-scores are almost less then 0.8. The performance has been improved as the model has been heavy. The DeepLabV3+^33^ gets higher point-level scores and boundary-level scores, especially in detecting straight-going sides. The performance of the HRNet-w18^32^ is the best not only on the point-level assessment but also on the boundary-level assessment. About three-quarters of the boundaries have been predicted with high point-level scores. More than half of the boundaries have a score larger than 0.8.

**Table 5:** Performance among detectors.

Backbone	Straight-going side				Turning				Ignoring semantics			
	*mF*_*p*_	*F*_0.3_	*F*_0.5_	*F*_0.8_	*mF*_*p*_	*F*_0.3_	*F*_0.5_	*F*_0.8_	*mF*_*p*_	*F*_0.3_	*F*_0.5_	*F*_0.8_
UNet^31^	0.55	0.50	0.41	0.07	0.71	0.68	0.63	0.41	0.75	0.70	0.67	0.32
HRNet-w18^32^	**0.69**	**0.66**	**0.63**	**0.56**	**0.73**	**0.75**	**0.69**	**0.54**	**0.82**	**0.83**	**0.77**	**0.62**
DeepLabV3+^33^	0.62	0.57	0.49	0.35	0.71	0.67	0.61	0.43	0.78	0.73	0.67	0.46

*mF*_*p*_ is the average point-level F1-scores.

**Fig. 12:**
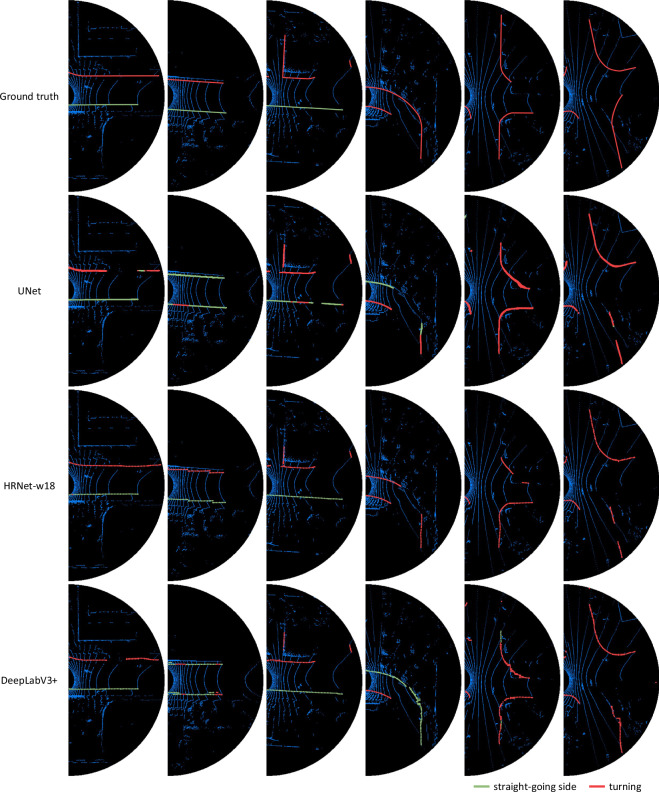
Visualized results of the ground truth, UNet^31^, HRNet-w18^32^ and DeepLabV3+^33^.

Some results are visualized in Fig. 12. The UNet^31^ has poor cognition of the integrity of a boundary, and it is easy to divide a complete boundary into multiple parts, which may be caused by shallow perception. Compare to the HRNet-w18^32^, the DeepLabV3+^33^ is poor recognition on guiding semantics and has insufficient grasp of detailed features. The frequent exchanges between multiple scales in the HRNet-w18^32^ may have a significant impact. The three models have trouble in dealing with occlusion and curves. The cognition of the integrity of boundaries and a scene needs to be enhanced. Although the performance can be further improved, the results show that the TAB^14^ can support training and evaluating travelable area boundary detection models.

### Importance of scene complexity labels

Scene complexity labels mark the parts affected by the diversity of boundaries, the interaction between the robot and objects and the sparsity of point clouds. They directly play an important role in calculating tolerant radii when judging which predicted points are correct during evaluation. These variable tolerant radii result from the consideration of task requirements and the tolerance for annotation error. On the other hand, they have a direct impact on supervision in the supervised training phase as illustrated in equation (8).

Here, the HRNet-w18^32^ is used as a backbone to detect travelable area boundaries and verify the importance of scene complexity labels. Two detectors are trained. When training the first detector, the tolerant radii are variable as illustrated in equation (3), while when training another detector, the tolerant radii are fixed and set as *r*_*base*_. During evaluation, the tolerant radii are variable or fixed as *r*_*base*_ too. Table 6 shows the performance of the two detectors evaluated with variable or fixed tolerant radii. Variable tolerant radii during training can reduce false positives as the most *mF*_*p*_ and *F*_0.3_ are higher than those trained with fixed tolerant radii. On the other hand, the scores are decreased when the detectors are evaluated with fixed tolerant radii. In a word, it is beneficial for a detector to take scene complexity labels into account during training and evaluation.

**Table 6:** Importance of scene complexity labels.

Evaluator	Detector	Straight-going side				Turning				Ignoring semantics			
		*mF*_*p*_	*F*_0.3_	*F*_0.5_	*F*_0.8_	*mF*_*p*_	*F*_0.3_	*F*_0.5_	*F*_0.8_	*mF*_*p*_	*F*_0.3_	*F*_0.5_	*F*_0.8_
Variable	Variable	0.69	0.66	0.63	0.56	0.73	0.75	0.69	0.54	0.82	0.83	0.77	0.62
	Fixed	0.67	0.66	0.62	0.54	0.75	0.73	0.68	0.54	0.83	0.81	0.76	0.62
Fixed	Variable	0.67	0.65	0.62	0.55	0.65	0.70	0.62	0.42	0.75	0.79	0.71	0.53
	Fixed	0.66	0.65	0.62	0.52	0.68	0.70	0.63	0.43	0.77	0.78	0.72	0.53

The tolerant radii are variable or fixed during training or evaluation. *mF*_*p*_ is the average point-level F1-scores.

## Usage Notes

The proposed TAB^14^ has great potential for visual navigation. It provides guiding semantics of travelable area boundaries and can promote the development of new algorithms for both road boundary detection and travelable area boundary detection. Researches on robust detectors are expected. Visual navigation systems could be developed based on guiding semantics. End-to-end navigation could be improved with the help of guiding semantics.

Due to the fact that the selected scenes cannot cover all the diverse roads and traffic environments, applications developed based on this dataset are recommended to be further adapted, including transfer learning and parameter tuning using data collected from target scene.

**Fig. 13:**
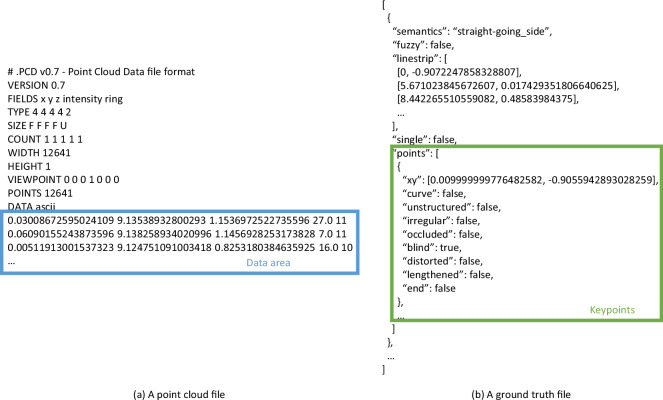
Examples of provided point cloud files and ground truth files.

As illustrated in Fig. 13, the provided point cloud files are in PCD format and the ground truth files are in JSON format. PCD readers and JSON readers can be used to load and read the files. Or the files can be read as text files. The data in the data area as shown in Fig. 13(a) are points in a point cloud frame. One line records a point and has 5 numbers including the 3D coordinates (*x*, *y*, *z*) in the point cloud space, the reflection intensity and the index of beam. The data encircled by the green rectangular is keypoints of a boundary in Fig. 13(b).

A Python toolkit (https://github.com/kaiopen/tab_kit) for quick access to the TAB^14^ is provided. After preparing the dataset, input the path to the dataset to the toolkit. Both point clouds and labels can be easily accessed via just one line of code. The frames in a sequence can be obtained chronologically through a loop so that detectors using temporal data can be developed. The provided point clouds are well adjusted and clipped, and can be directly fed into detectors. Labels and prediction results can be visualized via the toolkit. The interface of an evaluation method is provided too. Some examples have been released and are available along with the toolkit. With the help of the toolkit, point clouds can be loaded and fed into the detectors mentioned in the experiments. And then boundaries with their guiding semantics can be detected.

## Data Availability

The TAB^14^ has been released and available. The source codes for processing raw data, annotation, analysis and visualization are available in the GitHub repository (https://github.com/kaiopen/tab_creator). The codes to train, evaluate and test the travelable area boundary detectors, as well as the three well-trained models, are available in the GitHub repository (https://github.com/kaiopen/tabdet). The Python toolkit for quick access to the TAB^14^ is provided in the GitHub repository (https://github.com/kaiopen/tab_kit) too.
